# Excited-state intramolecular proton transfer with and without the assistance of vibronic-transition-induced skeletal deformation in phenol–quinoline

**DOI:** 10.1039/d1ra07042h

**Published:** 2021-11-19

**Authors:** Yu-Hui Liu, Shi-Bo Yu, Ya-Jing Peng, Chen-Wen Wang, Chaoyuan Zhu, Sheng-Hsien Lin

**Affiliations:** College of Physical Science and Technology, Bohai University Jinzhou 121013 China yhliu@bhu.edu.cn; Department of Applied Chemistry and Institute of Molecular Science, National Chiao-Tung University Hsinchu 30010 Taiwan cyzhu@mail.nctu.edu.tw; Department of Applied Chemistry and Center for Emergent Functional Matter Science, National Yang Ming Chiao Tung University Hsinchu 30010 Taiwan

## Abstract

The excited-state intramolecular proton transfer (ESIPT) reaction of two phenol–quinoline molecules (namely PQ-1 and PQ-2) were investigated using time-dependent density functional theory. The five-(six-) membered-ring carbocycle between the phenol and quinolone moieties in PQ-1 (PQ-2) actually causes a relatively loose (tight) hydrogen bond, which results in a small-barrier (barrier-less) on an excited-state potential energy surface with a slow (fast) ESIPT process with (without) involving the skeletal deformation motion up to the electronic excitation. The skeletal deformation motion that is induced from the largest vibronic excitation with low frequency can assist in decreasing the donor–acceptor distance and lowering the reaction barrier in the excited-state potential energy surface, and thus effectively enhance the ESIPT reaction for PQ-1. The Franck–Condon simulation indicated that the low-frequency mode with vibronic excitation 0 → 1′ is an original source of the skeletal deformation vibration. The present simulation presents physical insights for phenol–quinoline molecules in which relatively tight or loose hydrogen bonds can influence the ESIPT reaction process with and without the assistance of the skeletal deformation motion.

## Abstract 2

Skeletal deformation motion is demonstrated from the specific vibronic excitation of phenol–quinoline molecules.

## Graphical abstract

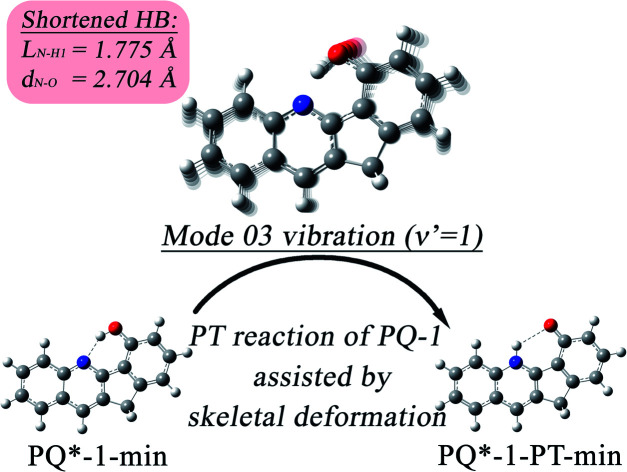

## 1. Introduction

Excited-state intramolecular proton transfer (ESIPT) reaction dynamics *via* intermolecular and/or intramolecular proton transfer have been extensively investigated based on accurate theoretical methods and advanced experimental technologies.^1–14^ The ESIPT mechanism is of fundamental importance and has been widely applied to many fields such as optical materials, fluorescence sensors, and chemistry and biology.^15–23^

The ESIPT reaction dynamics described by the well-known Eigen–Weller model can be classified as a two-step process.^24,25^ The first step is a short-range proton transfer that forms a contact ion pair upon excitation, and the second step is separation of the contact ion pair into free ions. Recently, the validity of the estimated rate constants based on the Eigen–Weller model has been reasonably doubted,^26,27^ and this has occurred because many more complicated ESIPT mechanisms exist that can be roughly divided into three classes.^28,29^ The proton-transferring model is a proton-tunneling model with a strong isotope effect,^30–32^ whereby protons are transferred along a shortened donor–acceptor path by skeletal deformation motion induced from low-frequency vibration,^33–36^ and there is an intermediate mechanism between these two models.

The hydrogen bond has played a very important role in increasing our understanding of the reaction mechanism in photochemistry and photophysics over the past decades. Han and Zhao^37–40^ proposed an excited-state strengthened hydrogen bond theory that was observed in experiments. Hydrogen bonds actually act as a proton transfer pathway for the ESIPT reaction dynamics, and their dynamic influence can be further enhanced in the excited state rather than in the ground state.^41–45^ It is worthwhile to investigate similar molecules to determine if ESIPT mechanisms prefer proton-tunneling or skeletal deformation in relation to hydrogen bond lengths (or bonding strengths). However, this type of contrastive investigation is rare due to the lack of suitable molecular systems for the comprehensive understanding of such ESIPT mechanisms.

Recently, Parada and co-workers^46^ synthetized a series of phenol–quinoline (PQ) compounds for the purpose of studying ESIPT mechanisms with different proton donor–acceptor distances and different dihedral angles between the phenol and quinolone moieties. They found that a longer donor–acceptor distance could lead to a higher barrier for ESIPT reactions, and the nonplanar structure could promote deactivation after the proton transfer process in the S_1_ state without the formation of a tautomer. They commendably revealed the important role of ESIPT reaction mechanisms in relation to hydrogen bond length (or donor–acceptor distance), but they could not relate its mechanisms to either proton-tunneling or the skeletal deformation motion. Actually, PQ-1 (associated with a five-member-ring carbocycle) and PQ-2 (associated with a six-member-ring carbocycle) with different hydrogen-bond lengths have been perfect molecules for revealing the relationship between the hydrogen bond (tight or loose) and the proton transfer characteristics. Moreover, it has been more recently demonstrated that other relevant factors can also act together with hydrogen bonds in the ESIPT reaction, such as the p*K*_a_ of the OH, the electron density shape along OH–N, and the symmetry of the aromatic orbitals.^47,48^

In the present study, we investigated PQ-1 and PQ-2 in detail to theoretically examine their ESIPT mechanisms in relation to different hydrogen-bond lengths by using density functional theory (DFT) and time-dependent (TD-DFT) methods. The PQ-3 molecule associated with a seven-member-ring carbocycle between the phenol and quinolone was not studied in this work because of its totally different nonplanar effect. The electronic structural optimization showed that hydrogen bonds are shorter and tighter in PQ-2 than in PQ-1.

We analyzed the coordination of skeletal deformation vibrations (which distinctly decrease the donor–acceptor distance) with hydrogen bond lengthening (the proton transfer pathway) to confirm the skeletal deformation ESIPT mechanism in the S_1_ state. As is well known, the higher vibrational quantum numbers would lead to a larger displacement from the equilibrium position. Hence, the Franck–Condon factors for the vibronic transitions are calculated within the displaced harmonic approximation for the purpose of discussing the vibronic transition distributions in the vibrational excited modes upon photoexcitation, so that they can more effectively promote the proton transfer reaction.

## 2. Computational methods and details

Ground-state (excited-state) geometry optimizations of PQ-1 and PQ-2 were performed using (TD)-DFT with the (TD)-B3LYP^49,50^ functional and basis set of triple-ζ valence quality with one set of polarization functions (TZVP^51^) throughout the present work. In addition, the dielectric constant *ε* = 35.688 in Gaussian for acetonitrile solvent was selected in all calculations within the polarizable conductor calculation model (CPCM).^52,53^ The potential energy surfaces (PESs) were constructed by a series of single-point energy calculations for the structures manually modified. For PQ-1, as a PT dimension, the N–H1 was lengthened from 0.9 to 2.2 Å with a step size of 0.1 Å. For the vibrational dimension, we modified the structure along the vibrational direction from 0.1 to −1.2 Å with a step size of 0.1 Å. Hence, 168 (12 × 14) points were included for constructing the PES of PQ-1. In the case of PQ-2, N–H1 was lengthened from 0.9 to 1.8 Å, and the structure was modified along the vibrational direction from −0.1 to 1.0 Å. Thus, 120 (10 × 12) points were included for constructing the PES of PQ-2. All of these electronic structure calculations were carried out using the Gaussian 09 program suite.^54^

The Franck–Condon factor for the vibronic transition from the electronic-ground-state (a) vibrational *ν*_*i*_ = 0 level to the electronic-excited-state (b) vibrational 
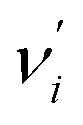
 level can be obtained within independent displaced harmonic approximation as:^55,56^1
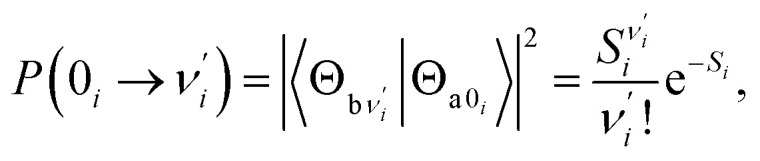
where 
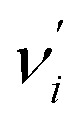
 denotes the vibrational quantum number of the *i*th normal mode in the electronic excited state, and *S*_*i*_ denotes the Huang–Rhys factor defined as:2
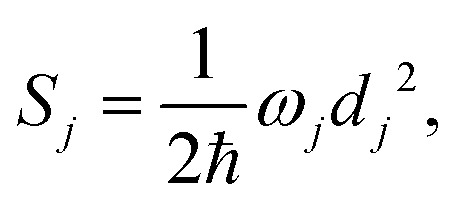
where the displacement *d*_*j*_ is given by:3
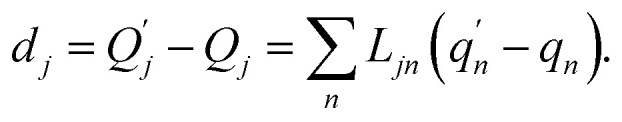


The 
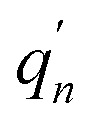
 and *q*_*n*_ in eqn (3) are the mass-weighted Cartesian coordinates at equilibrium geometries of the electronic excited and ground states, respectively. The transformation matrix *L* in eqn (3) can be computed with frequency analysis using the Gaussian program. The vibronic transition calculations were carried out using our own code.

## 3. Results and discussion

There are two options that can be used to describe the solvation effect in the TDDFT calculation: the linear response (LR) solvation model or the state-specific (SS) solvation model. The SS model can usually provide more accurate results than the LR model but requires longer computing time. However, because of the absence of the analytic gradient in the TDDFT method, the SS model is unsuitable for optimizing the excited-state structures. However, absorption (emission) is vertical electronic excitation (de-excitation) according to the Franck–Condon approximation, while the orientation motions of the solvent molecules are too slow to compare with electronic excitation (de-excitation). The initial (final) electronic state is (not) at the minimum of the potential energy surface, and thus, the initial (final) electronic state should be calculated with the equilibrium (nonequilibrium) solvation model as follows:

4 Absorption energy = *E*_ES_(*R*_GS_, non-EQ) − *E*_GS_(*R*_GS_, EQ)

and

5 Emission energy = *E*_ES_(*R*_ES_, EQ) − *E*_GS_(*R*_ES_, non-EQ),

where the subscript GS (ES) denotes the ground (excited) state, *R*_GS_ (*R*_ES_) denotes nuclear coordinates at the ground (excited) state potential energy minimum, and Eq (non-Eq) denotes the equilibrium (nonequilibrium) solvation model. This is the LR solvation model in the Gaussian program. Hence, the LR solvation model remains a satisfactory choice for the excited-state structure optimization with the available analytic gradient. The EQ and non-EQ settings are also in accord with the actual situation.

**Fig. 1:**
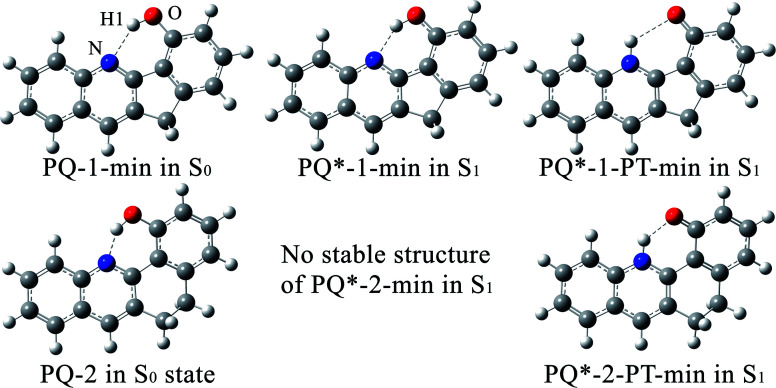
The optimized electronic structures at potential energy minima in the S_0_ and S_1_ states for PQ-1 and PQ-2 molecules.

**Fig. 2:**
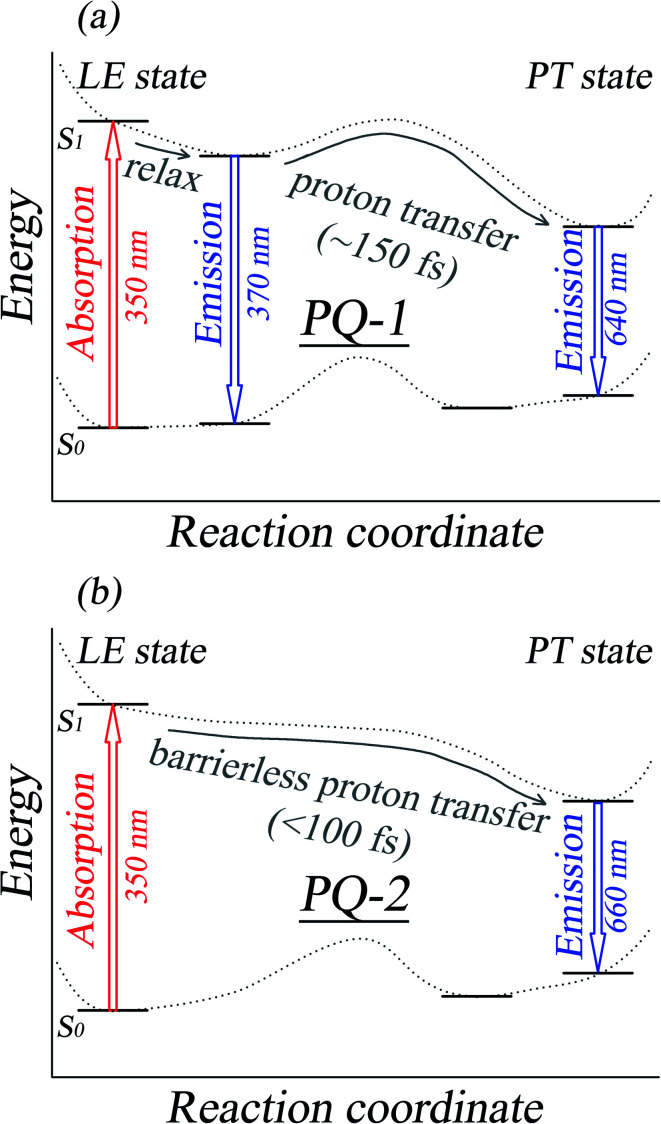
A schematic graph of the ESIPT mechanisms for PQ-1 and PQ-2, as well as the assignments for the absorption and fluorescence spectra. All the spectral data and the reaction rate constants were obtained from experimental measurements according to ref. 46.

Within the LR solvation model, the optimized electronic structures of PQ-1 and PQ-2 molecules in the acetonitrile solvent (used for the experimental measurement^46^) are shown in Fig. 1 with different hydrogen bonds (tight or loose) for the S_0_ and S_1_ states, respectively. We used PQ-1-min (PQ-2-min) to represent the global minimum at the Franck–Condon absorption region on ground state S_0_, as shown in Fig. 2a(b), and PQ-1-PT-min (PQ-2-PT-min) to denote the local minimum after proton transfer (PT) on ground state S_0_. All the energies of the stable structure in Fig. 2 have been corrected by zero-point vibration energy (ZPVE). There are double wells on the ground-state S_0_ potential energy surfaces for both PQ-1 and PQ-2 molecules. There are double wells (namely PQ*-1-min and PQ*-PT-1-min) on excited-state S_1_ for PQ-1, but there is only a single well (namely PQ*-2-PT-min) on excited-state S_1_ for PQ-2. The N–H1 bond length and the distance between N and O are 2.032 Å (1.671 Å) and 2.892 Å (2.583 Å), respectively for PQ-1-min (PQ-2-min), as summarized in Table 1. This indicates that the hydrogen bond is relatively longer and looser at PQ-1-min (due to the five-member-ring carbocycle between the phenol and quinolone) than at PQ-2-min (due to the six-member-ring carbocycle between the phenol and quinolone).

**Table 1:** Optimized key geometries (hydrogen bond length and donor–acceptor distance) at reactants PQ (PQ*)-1-min and PQ-2-min, and at products PQ*-1-PT-min and PQ*-2-PT-min

Bond length (Å)	PQ(PQ*)-1-min		PQ-2-min		PQ*-1-PT-min	PQ*-2-PT-min
	S_0_	S_1_	S_0_	S_1_	S_1_	S_1_
N–H1	2.032	1.860	1.671	—	1.014	1.021
O–H1	0.981	1.006	1.003	—	2.392	1.894
N–O	2.892	2.772	2.583	—	3.057	2.703

There is PQ*-1-min on the S_1_ state for the PQ-1 molecule, but there is no PQ*-2-min on the S_1_ state for the PQ-2 molecule, and this might be due to the tight hydrogen bond at PQ-2-min. Once it is vertically excited to the S_1_ state from PQ-2-min, it quickly decays to PQ*-2-PT-min with barrierless or a nearly barrierless proton transfer process, as observed in the experiment (the measured transfer time was <100 fs, as shown in Fig. 2b).^46^ This supports the conclusion that the tighter hydrogen bond can effectively facilitate the ESIPT reaction. However, there is PQ*-1-min on the S_1_ state for PQ-1 molecules, so that once it is vertically excited to the S_1_ state from PQ-1-min, it decays to PQ*-1-PT-min less quickly with a small barrier from PQ*-1-min for the proton transfer process, as observed in the experiment (a longer transfer time of approximately 150 fs was measured, as shown in Fig. 2a).^46^ The hydrogen bond in PQ-1 is relatively weaker than that in PQ-2 in the excited state, and Table 1 shows that the N–H1 bond length of 1.860 Å at PQ*-1-min on the S_1_ state is even longer than the corresponding bond of 1.671 Å at PQ-2-min on the S_0_ state. Thus, there should be a certain population at the PQ*-1-min potential energy well, although there are ESIPTs to the PQ*-1-PT-min energy well.

### 3.1. Simulated absorption and emission spectra

**Fig. 3:**
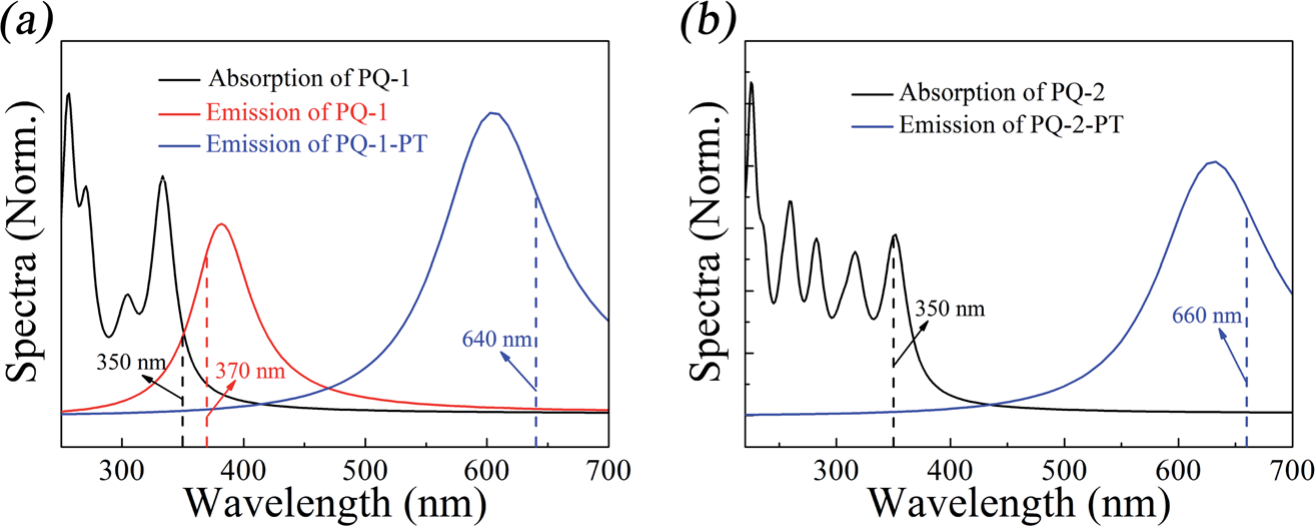
Simulated absorption and emission spectra for the PQ-1 and PQ-2 molecules, including the ‘abnormal’ emission from PQ*-1-PT and PQ*-2-PT. The vertical dashed lines indicate the corresponding experimental spectral peak positions for PQ-1 and PQ-2 in acetonitrile.^46^

We first simulated the absorption spectra at PQ-1-min (PQ-2-min) in the Franck–Condon region, vertically excited up to the 20th singlet excited state as shown. The simulated absorption spectral profiles (with oscillator strengths serving as relative intensities) are plotted in Fig. 3a(b) for the PQ-1 (PQ-2) molecule. The simulated first absorption peak at 334 nm (352 nm) for the S_1_ state is in agreement with the experimental value of 350 nm (350 nm), as shown in Fig. 3a(b) for the PQ-1 (PQ-2) molecule. However, simulated high-level absorption spectrum profiles oscillate much higher than the sixth singlet excited state, with large oscillator strengths, as shown in Fig. 3. The simulated emission spectrum at PQ*-1-PT-min (PQ*-1-min) with vertical de-excitation to the ground state exhibits emission peaks at 605 nm (385 nm) that are in agreement with the experimentally measured value of 640 nm (370 nm), as shown in Fig. 3a, in which there are overlapping regions between the two emission bands. This confirms that experimentally observed emission spectra must originate from a mixed contribution before and after the ESIPT process in the S_1_ state for the PQ-1 molecule. However, there is an emission peak at 631 nm for the simulated emission spectrum at PQ*-2-PT-min, which is in satisfactory agreement with the experimental value of 660 nm, as shown in Fig. 3b, in which the experimentally observed emission spectra are only contributed after the ESIPT process occurs in the S_1_ state for the PQ-2 molecule.

**Fig. 4:**
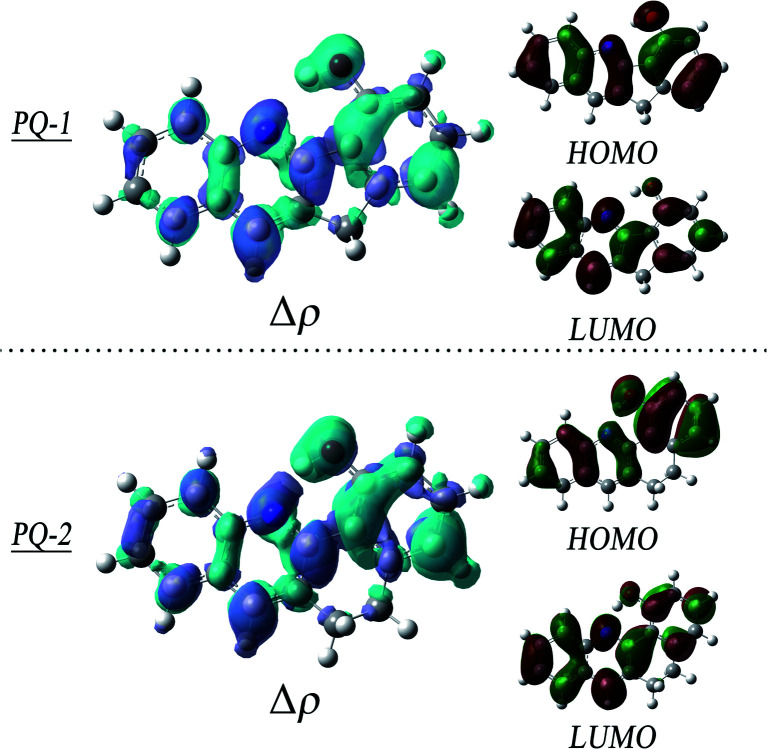
Plots of the electron density change upon excitation (Δ*ρ* = *ρ*_S1_ − *ρ*_S0_, isovalue = 0.001), and Frontier molecular orbitals (MOs). The upper panel is at PQ-1-min, and the lower panel is at PQ-2-min.

To obtain insights into the electronic transitions and the nature of the excited states, the Frontier molecular orbitals (highest occupied molecular orbital (HOMO) and lowest unoccupied molecular orbital (LUMO)) are shown in Fig. 4, accompanied by plots of the electron density change upon excitation. It can be clearly seen from the molecular orbitals that the π character of the HOMO and the π* character of the LUMO are the almost same as the ππ* excitation at QP-1-min and QP-2-min. The changes in the density on the proton donor (OH) group and the proton acceptor (N atom) are visible in both species, which is more remarkable in PQ-2 and leads to the barrierless ESIPT reaction.

### 3.2. Vibronic excitation analysis of the ESIPT process

The ESIPT process leads to a decrease in the donor–acceptor distance so that the reaction barrier is lower in the excited state, and thus, the potential energy well on the S_1_ state can only support vibrational states with smaller vibrational frequencies, which correspond to the skeletal deformation motion. This skeletal deformation actually assists the proton transfer reaction in the ESIPT process. We first compared the time scale of intramolecular vibrational redistribution (IVR), in which vibrationally excited energies are redistributed into many different vibrational states. This time scale is 10^3^–10^6^ fs, but the decay time is approximately 150 fs for the PQ-1 ESIPT process from PQ*-1-min (observed emission at 370 nm in Fig. 2a) to PQ*-1-PT-min (observed emission at 640 nm in Fig. 2a). The decay time in the ESIPT process is much faster than the IVR time scale.

It is likely that the ESIPT process must be assisted by a particular excited vibronic mode in which its energy is not dissipated. We utilized a vibronic excitation analysis method to determine which vibronic mode is responsible for skeletal deformation motion and to understand how this mode can assist the proton transfer reaction. The largest 
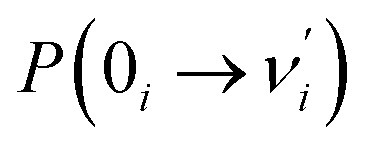
 value in eqn (1) denotes that the *i*-th mode is mostly an excited vibronic mode in light absorption from the electronic ground to the excited state, and thus, this *i*-th mode corresponds to skeletal deformation motion. As is well known, the largest Huang–Rhys factor *S*_*i*_ yields the largest 
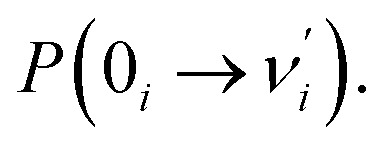


**Fig. 5:**
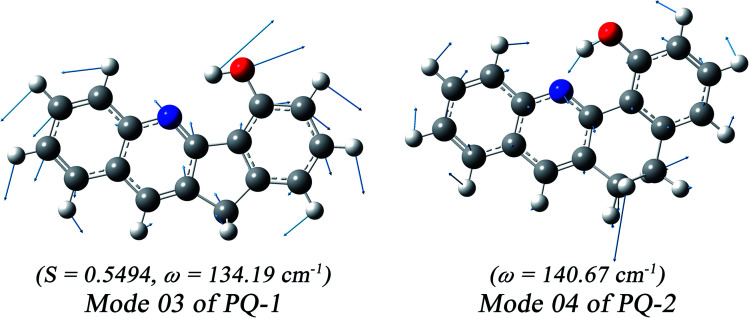
The largest *S* value was obtained for calculated vibronic mode 03 with a vibrational frequency of 134.19 cm^−1^ at PQ-1-min and similar mode 04 with vibrational frequency of 140.67 cm^−1^ at PQ-2-min.

**Fig. 6:**
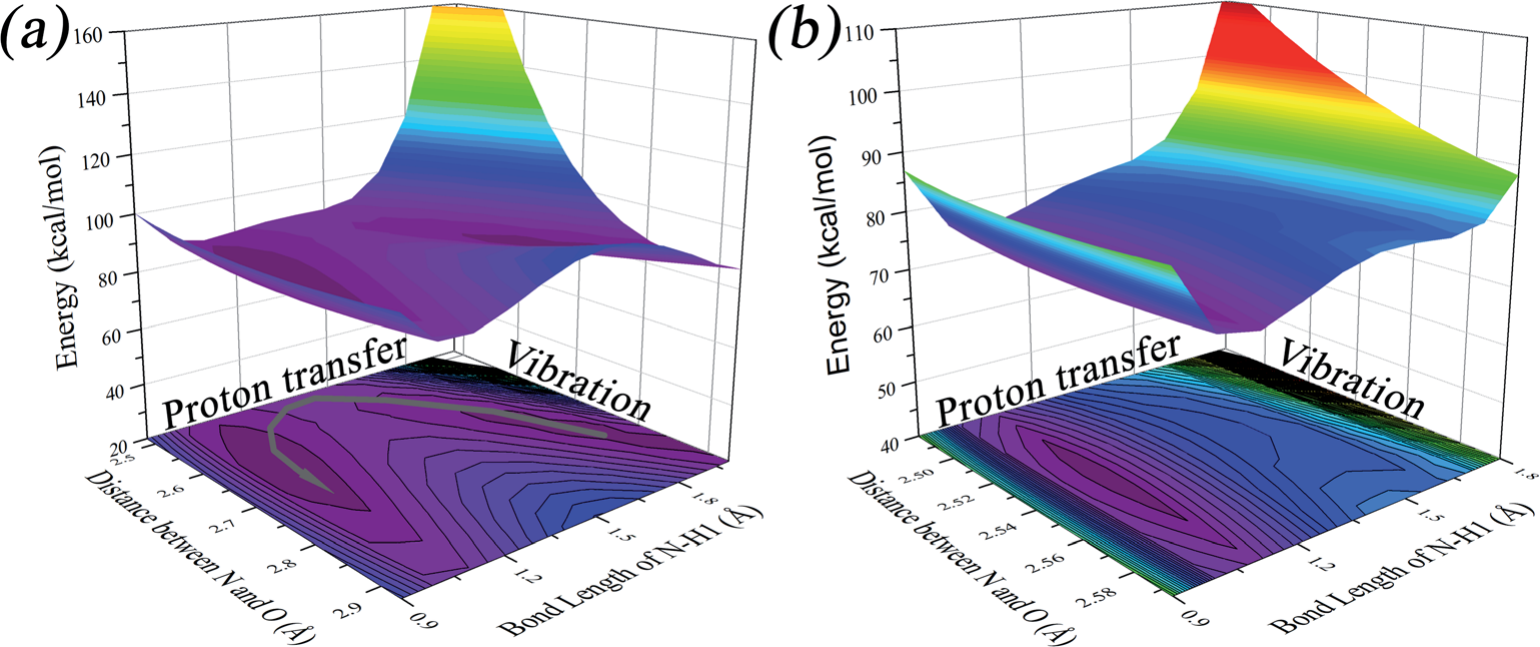
Two-dimensional potential energy surfaces on the S_1_ state along N–H1 length and vibrational mode 03 (04) for PQ-1 (PQ-2), which corresponds to the skeletal deformation motion only for PQ-1. (a) The PQ-1 molecule and (b) PQ-2 molecule.

We found that one mode with a low vibrational frequency of 134.19 cm^−1^ exhibited the largest *S* = 0.5494 (*S* ∼ 10^−2^ for the other frequencies lower than 600 cm^−1^) for the PQ-1 molecule. This mode was named 03 for its corresponding vibrational motion pattern, as shown in the left panel of Fig. 5. Mode 03 corresponds to the skeletal deformation that assists in decreasing the N–O distance in the ESIPT process. Next, we drew two-dimensional (2D) potential energy surfaces for the S_1_ state along vibrational mode 03 (the skeletal deformation motion) and bond length N–H1, as shown in Fig. 6a, in which the potential energy barrier was reduced to as low as 9.42 kcal mol^−1^. This further confirms that mode 03 as a skeletal deformation motion is a unique choice for assisting and enhancing the ESIPT reaction for the PQ-1 molecule. Moreover, the molecule would first reach the LE state upon excitation, as shown in Fig. 2, where the energy is much higher than the equilibrium geometry in the S_1_ state. The excess energy released in the LE state relaxes the equilibrium geometry to assist the reactant molecules in climbing the energy barrier. The excess energy (*E*_LE_ − *E*_eq_) was calculated to be 9.74 and 6.50 kcal mol^−1^ with and without the ZPVE correction, which can effectively accelerate the ESIPT reaction.

For comparison with PQ-1, we discussed that the ESIPT process leads to a decrease in the donor–acceptor distance so that the reaction barrier is even lower or the barrier is less in the excited state before proton transfer for the PQ-2 molecule. Actually, there is no potential energy barrier along the ESIPT pathway, as shown in Fig. 2b. Therefore, there is no potential well the in S_1_ state to support any vibrational state, and thus, that particular vibronic excitation analysis does not seem applicable. Because PQ-2-min is similar to PQ-1-min on ground S_0_ state, we simply utilized a similar vibrational motion pattern for mode 03 in PQ-1-min, as shown in the right panel of Fig. 5, and a vibrational frequency 140.67 cm^−1^ for the PQ-2 molecule, namely as mode 04, was measured for this similar mode. Mode 03 in PQ-1 is a vibrational motion pattern similar to that of mode 04 in PQ-2, but it is simply an opposite vibrational direction. We drew 2D potential energy surfaces for the S_1_ state again along vibrational mode 04 and bond length N–H1, as shown in Fig. 6b, in which there is no potential energy barrier as expected. Therefore, we concluded that the ESIPT for PQ-2 is not related to skeletal deformation motion, but rather, it is a simple switching of electronic orbitals due to the tighter hydrogen bond.

**Fig. 7:**
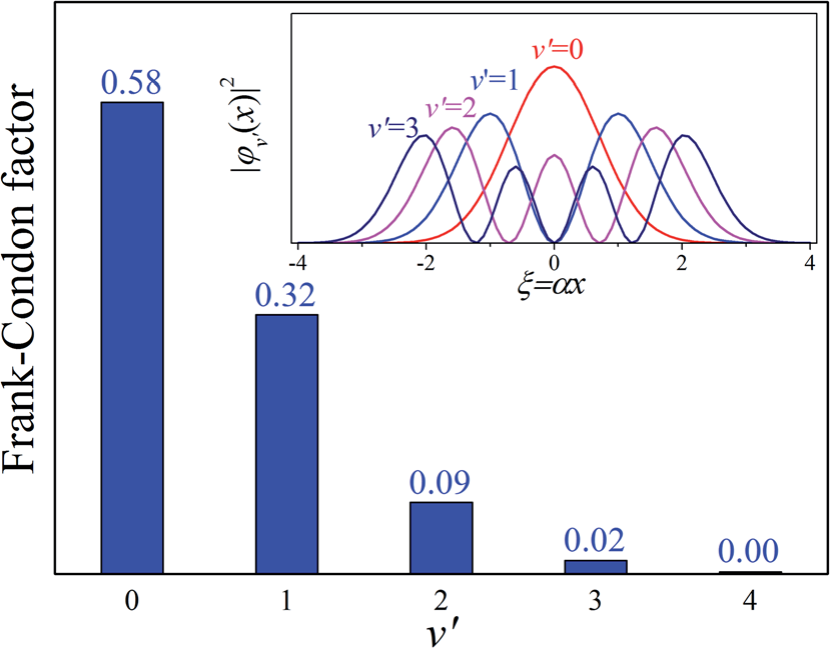
Calculated Franck–Condon distributions 
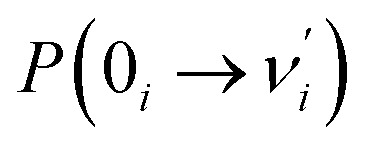
 of the vibronic transition of mode 03 for the PQ-1 molecule. The inset shows the probability distributions of the one-dimensional harmonic oscillator with the vibrational quantum number (*v*′ = 1, 2, 3, and 4), where *α* = (*mω*/*ħ*)^1/2^.

**Fig. 8:**
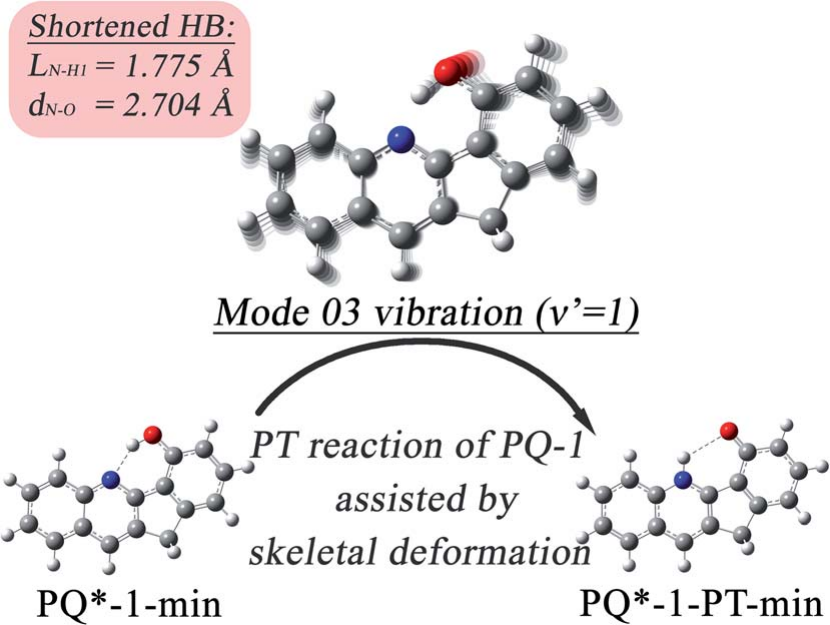
The skeletal deformation motion is pictured as slip shots of vibrational (*v*′ = 1 in mode 3) animation motion for the PQ-1 molecule.

Our analysis indicated that vibronic transition mode 03 is the skeletal deformation motion that is responsible for assisting ESIPT from PQ*-1-min, and it would be interesting to determine the vibronic excitation distribution 
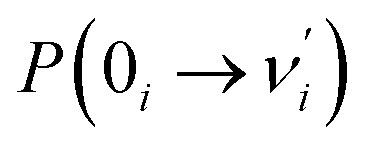
 for mode 03 to understand which specific vibronic excitation level contributes the most. We used eqn (1) to obtain *P*(0 → 0′) = 0.58 and *P*(0 → 1′) = 0.32 , as shown in Fig. 7, so that the 0 → 0′ and 0 → 1′ vibronic excitations contributed the most for mode 03. However, we need the probability distribution (vibration wave function square) to be expressed in terms of the displacement to the equilibrium position (*ξ* = 0 in Fig. 7), for which it is in the direction moving forward to assist ESIPT. Fig. 7 indicates that there is a much larger probability for 0 → 1′ than 0 → 0′ at the large displacement to *ξ* = 1. Finally, we concluded that the vibronic 0 → 1′ excitation of mode 03 actually corresponds to the skeletal deformation motion that assists the ESIPT from PQ*-1-min to PQ*-1-PT-min in the S_1_ state. This skeletal deformation motion appears as slip shots of vibrational (*v*′ = 1 in mode 3) animation motion, as shown in Fig. 8.

At the displacement *ξ* = 1, where vibrational motion has the maximum probability for *v*′ = 1 in mode 03, this displacement from a normal-mode coordinate is 0.211 Å away from the equilibrium position. The N–O distance and the hydrogen bond N⋯H1 length have been decreased to 2.704 Å and 1.775 Å, respectively, at *ξ* = 1 (in comparison with N–H1 = 1.860 Å at *ξ* = 0 equilibrium) in the S_1_ state. Therefore, the hydrogen bond becomes tighter with the assistance of the excited vibration *v*′ = 1 in mode 03, as shown in Fig. 8. However, the original tight hydrogen bond in the PQ-2 molecule (N–H1 = 1.671 Å at *ξ* = 0 equilibrium in the S_0_ state) can lead to a direct ESIPT reaction without any assistance from the skeletal deformation motion.

## 4. Concluding remarks

We performed quantum chemistry calculations at the TD-DFT level to investigate the ESIPT reaction mechanism for the PQ-1 and PQ-2 molecules. We found that tighter hydrogen bonds led to a faster ESIPT process in the electronically excited state, as experimentally observed. Hydrogen bonds were tighter/shorter in the PQ-2 molecule than in PQ-1, and this was due to the existing six (five)-member-ring carbocycle between the phenol and quinolone for PQ-2 (PQ-1). Therefore, tightened hydrogen bonds in PQ-2 resulted in a barrier-less potential energy surface with a fast ESIPT process without involving the proton-tunneling model or skeletal deformation motion during the electronic excitation. In contrast, a relatively loose hydrogen bond in PQ-1 resulted in a small barrier potential energy surface with a relatively slow ESIPT process involving skeletal deformation motion up to the electronic excitation.

This skeletal deformation motion that corresponds to vibronic excitation with mode 03 actually assisted in decreasing the donor–acceptor (N–O in this case) distance and lowering the reaction barrier in the S_1_ potential energy surface, and thus, it effectively enhanced the ESIPT reaction. More specifically, it is mode 03 with vibronic excitation 0 → 1′ that corresponds to the skeletal deformation motion for enhancing the ESIPT reaction for the PQ-1 molecule. The present study provides physical insights for many other structurally similar systems in which tight or loose hydrogen bonds can influence the ESIPT reaction process with and without assistance from skeletal deformation motion. This skeletal deformation motion originates from the strongest vibronic transition up to photoexcitation.

## Conflicts of interest

The authors declare no competing financial interests.

## Supplementary Material
